# Systemic Immune Inflammation Index as a Key Predictor of Dialysis in Pediatric Chronic Kidney Disease with the Use of Random Forest Classifier

**DOI:** 10.3390/jcm12216911

**Published:** 2023-11-03

**Authors:** Anna Kawalec, Jakub Stojanowski, Paulina Mazurkiewicz, Anna Choma, Magdalena Gaik, Mateusz Pluta, Michał Szymański, Aleksandra Bruciak, Tomasz Gołębiowski, Kinga Musiał

**Affiliations:** 1 Department of Pediatric Nephrology, Wroclaw Medical University, Borowska 213, 50-556 Wroclaw, Poland; 2 Department of Nephrology and Transplantation Medicine, Wroclaw Medical University, Borowska 213, 50-556 Wroclaw, Poland; 3 Clinic of Pediatric Nephrology, University Clinical Hospital, Borowska 213, 50-556 Wroclaw, Poland; 4 Students’ Scientific Association, Department of Pediatric Nephrology, Wroclaw Medical University, Borowska 213, 50-556 Wroclaw, Poland

**Keywords:** chronic dialysis, chronic kidney disease, children, artificial intelligence, machine learning, systemic immune inflammation index

## Abstract

Background: Low-grade inflammation is a significant component of chronic kidney disease (CKD). Systemic immune inflammation index (SII), a newly defined ratio combining neutrophil, lymphocyte, and platelet counts, has not yet been evaluated in the pediatric CKD population nor in the context of CKD progression or dialysis. Thus, this study aimed to analyze the complete blood cell count (CBC)-driven parameters, including SII, in children with CKD and to assess their potential usefulness in the prediction of the need for chronic dialysis. Methods: A single-center, retrospective study was conducted on 27 predialysis children with CKD stages 4–5 and 39 children on chronic dialysis. The data were analyzed with the artificial intelligence tools. Results: The Random Forest Classifier (RFC) model with the input variables of neutrophil count, mean platelet volume (MPV), and SII turned out to be the best predictor of the progression of pediatric CKD into end-stage kidney disease (ESKD) requiring dialysis. Out of these variables, SII showed the largest share in the prediction of the need for renal replacement therapy. Conclusions: Chronic inflammation plays a pivotal role in the progression of CKD into ESKD. Among CBC-driven ratios, SII seems to be the most useful predictor of the need for chronic dialysis in CKD children.

## 1. Introduction

Persistent low-grade inflammation is a hallmark of chronic kidney disease (CKD) [1,2,3]. The coexisting immune dysfunction is a combination of chronic immune activation, resulting in a persistent pro-inflammatory state, and chronic immune suppression, leading to increased susceptibility to infections and poor vaccination response [1]. Such ambiguity is typical for many processes engaged in the CKD progression.

The increasing body of evidence suggests that the advanced production and decreased clearance of pro-inflammatory cytokines, as well as oxidative stress, are major causes of chronic inflammation under the conditions of impaired renal function [1,4,5]. Other factors contributing to the constant immune activation are metabolic acidosis, chronic and recurrent infections, dysbiosis of gut microbiota, changes in adipose tissue metabolism, and dialysis [1,3,6,7,8]. Immunocompetent cells also take part in a complex set of interactions with cell-derived chemokines, adhesive proteins, growth factors, or immune mediators responsible for the pro-inflammatory milieu [9]. 

Among the above-mentioned components, platelets show the widest panel of activity due to the multiplicity of active substances released from the granules, stimulating leukocyte migration, reactive oxygen species (ROS) generation, phagocytosis, and thrombotic events [9]. The platelet-derived inflammatory mediators interact with monocytes, neutrophils, and lymphocytes, promoting their recruitment and adhesion and upregulating the continued release of the proinflammatory mediators [9]. The diversity of lymphocyte subsets, ranging from pro-inflammatory to ambiguous to protective roles, adds to this puzzle [10]. Last but not least, uremic toxins and microbiota-derived metabolites create pro-inflammatory conditions, altering cellular immune responses [11].

The engagement of such a spectrum of cell subsets in CKD-derived complications has initiated interest in their potential applicability as universally available biomarkers of systemic inflammation. Therefore, recent research has focused on the analysis of complete blood cell count (CBC) parameters and their derivatives in patients with CKD. 

Differences in the pattern of total and differential leukocyte count have been observed when comparing adult CKD patients with non-CKD counterparts and pre-dialysis with dialysis adults [12,13,14,15,16,17]. The modulating role of platelets in CKD-related inflammatory pathways has also been reviewed [18]. Consequently, changes in the proportion of leukocyte subpopulations, platelet count, and mean platelet volume (MPV) have been linked with CKD progression in adults and proposed as biomarkers of inflammation [17,19,20,21,22,23,24]. 

The most recent studies have focused on the systemic immune inflammation index (SII), a newly defined ratio combining neutrophil, lymphocyte, and platelet counts. It is suggested as a prognostic indicator, comprehensively reflecting the inflammatory and immune status of patients with various renal diseases [25,26,27,28,29,30,31,32,33]. The data on SII in adults with CKD are limited, though elevated SII seems to have a predictive value for mortality risk among pre-dialysis and hemodialysis adult patients [30,31]. 

However, SII was not evaluated in the pediatric CKD population nor in the context of CKD progression or outcome, such as the need for chronic dialysis. Moreover, despite the increasing role of machine learning methods in clinical practice, they have not been used in the analysis of CKD-related inflammation so far. 

## 2. Aim of Study

Thus, this study aimed to analyze the complete blood cell count (CBC)-driven parameters, including SII, in children with CKD and to assess their potential usefulness in the prediction of the need for chronic dialysis, confronted with the predictive abilities of other indices of CKD-related complications, with the use of artificial intelligence tools. 

## 3. Materials and Methods

### 3.1. Patients’ Data

The study group consisted of 27 pre-dialysis children with CKD stages 4–5 (stage 4–11 patients, stage 5–16 children) and 39 patients on chronic dialysis (HD–20 children, APD–19 patients). The patients’ age ranged from 5 to 18 years; children under 5 were excluded due to the different CBC profiles regarding neutrophil–lymphocyte proportions. The evaluated CBC parameters were hemoglobin, hematocrit, leukocyte, neutrophil, monocyte, lymphocyte, platelet counts, and mean platelet volume (MPV). CBC-driven ratios (neutrophil-to-lymphocyte ratio (NLR), lymphocyte-to-monocyte ratio (LMR), platelet-to-lymphocyte ratio (PLR), and systemic immune inflammation index (SII = [neutrophil count-lymphocyte count]/platelet count)) were then calculated and analyzed. Kidney function (serum creatinine, eGFR) served as a direct classifier of patient subgroups (pre-dialysis, dialysis). The selected biochemical parameters (e.g., serum Ca, P, alkaline phosphatase, parathormone, cholecalciferol, transaminases, albumins, CRP) and blood gases were also evaluated. This database was used for further machine learning (ML) analysis.

### 3.2. Classical Statistical Analysis

Results were expressed as minimal, mean, maximal values, and standard deviation. The null hypothesis of normality of distribution of analyzed variables was rejected by Shapiro–Wilk test. Thus, the analysis was performed with the use of nonparametric Mann–Whitney U test. Correlations were assessed by Spearman’s rank correlation coefficient. Statistical analysis was performed using the package Statistica ver. 13.3 (StatSoft Inc., Tulsa, OK, USA). A *p* value < 0.05 was considered significant.

### 3.3. Machine Learning (ML) Statistical Scoring

Machine learning allows us to imitate simple decision-making processes and perform data classification based on previously calculated dependencies. These dependencies come from supervised learning, i.e., a person gives the correct labels for existing and available data, and the role of the program is to match the so-called hyperparameters to show the greatest possible discriminatory ability on the various input data that will be used to evaluate the models. Overcomplicating the model risks its overfitting to the data and ineffective prediction of the new data. Too little fit or too little complexity makes the model comparable to, or even worse than, the random classifier. An example of machine learning is the Random Forest Classifier, in which random decision trees are generated and then the so-called cost function is minimized in order to achieve the best fit in a compromise with flexibility. The final result is obtained by averaging the predictions from individual decision trees.

The small data size may be an obstacle to the use of machine learning. The main aspect limiting the application is the difficulty in training a model. In this study, the training on a small set of training data was effective, so we used several measures to describe the predictive properties. Additionally, the Random Forest Classifier is a relatively simple model used on small datasets by other authors, which justifies our attempt to use it [34].

The efficacy of the model can be characterized by its accuracy, area under the receiver operating curve (AUROC), precision, recall, F1-score, Matthews correlation coefficient (MCC), and Gini importance.

Accuracy is the ratio of correct matches or classifications to all classifications made, which indicates how close the model’s predictions are to the true value.

AUROC is the basic parameter defining the discriminating ability of the binary classifier.

Precision is an information technology synonym for a positive predictive value and is defined as the ratio of true positives to all positive predictions.

Recall is equivalent to sensitivity and determines the ratio of true positives to the sum of true positives and false negatives predictions.

F1-score is the harmonic mean of the precision and recall. F1-score is a stat especially useful in unbalanced sets due to classes. However, it has a limitation that can be circumvented by using a broader statistic, such as Matthews correlation coefficient (MCC). 

MCC is a parameter that achieves a large value if the model performs well enough in all four fields of the confusion matrix [35]. The use of all statistics extends the evaluation of many models but strengthens the proof of the effectiveness of the predictive model. 

The Gini feature importance determines how much a given feature contributes to dividing the set into classes. In other words, it is approximated by the probability of achieving a division that will allow the final classes to clarify themselves faster.

The aim was to narrow the set of input parameters so that the high predictive power is secured and overfitting or overcomplicating could be avoided. Moreover, the Gini importance was measured in order to define the parameter with the largest share in the prediction.

The primary database was randomized into a training and testing set in the ratio of 80:20, which is a proportion of the split commonly used and accepted in machine learning [36]. Due to the small size of the training set, we focused on 5-cross validation, which, using the simple heuristic algorithm described below, allowed us to quickly find an optimal solution, i.e., a Random Forest Classifier model with a sufficiently high discriminating power.

### 3.4. Heuristic Algorithm

In the first step, a random forest model was built using all available parameters as input variables. Of note, serum creatinine and eGFR values were not included in the analysis because they primarily stratified the groups, and their implementation would oversimplify the model. The Gini feature importance was calculated, and the parameter with the smallest contribution to the prediction was chosen. In the next step, we built a model based on variables, among which we did not include the aforementioned parameter. In this way, we have iteratively reduced the number of input variables to 3. The algorithm stopped the reduction of the number of variables when the model quality deteriorated after reducing the input size. 

The analysis of generated decision trees was based on following the process of dividing the data set. This method can be used to discover new hypotheses or design algorithms. Within a single decision tree, the same variables are queried but with different cutoffs or in different variants, with previous and following conditions stored in the nodes. Finally, the partial results from all trees were collected, and the prevailing result has become the final one.

## 4. Results

### 4.1. CBC Values and Correlations with Clinical Parameters

The values of CBC parameters did not differ between pre-dialysis and dialysis groups, except for the lymphocyte count, which was significantly lower in children on chronic dialysis (*p* < 0.005). The significant correlations of CBC parameters concerned serum urea and all cell subtypes: erythrocytes (R = −0.31; *p* = 0.01), leukocytes (R = −0.33; *p* = 0.007), neutrophils (R = −0.31; *p* = 0.01), lymphocytes (R = −0.25; *p* = 0.04), monocytes (R = −0.34; *p* = 0.004), and platelets (R = −0.35; *p* = 0.003). Additionally, correlations with inflammation markers (CRP and LEU (R = 0.29, *p* = 0.02), CRP and NEU (R = 0.39, *p* = 0.001), MPV and serum albumin (R = 0.29, *p* = 0.02)) and bone turnover indices (alkaline phosphatase and LYM (R = 0.34, *p* = 0.009), MPV and cholecalciferol level (R = 0.33, *p* = 0.01)) were statistically significant. Consequently, CBC-driven ratios revealed significant correlations (Table 1), although there was no connection between CBC values or CBC-derived ratios and major determinants of kidney function-serum creatinine or eGFR.

### 4.2. Machine Learning Analysis

The database was randomly split into training and testing subsets (Table 2). The training set was used to develop the best model. The test set allowed us to evaluate this model on new data, and their statistics are presented in the following sections.

**Figure 1:**
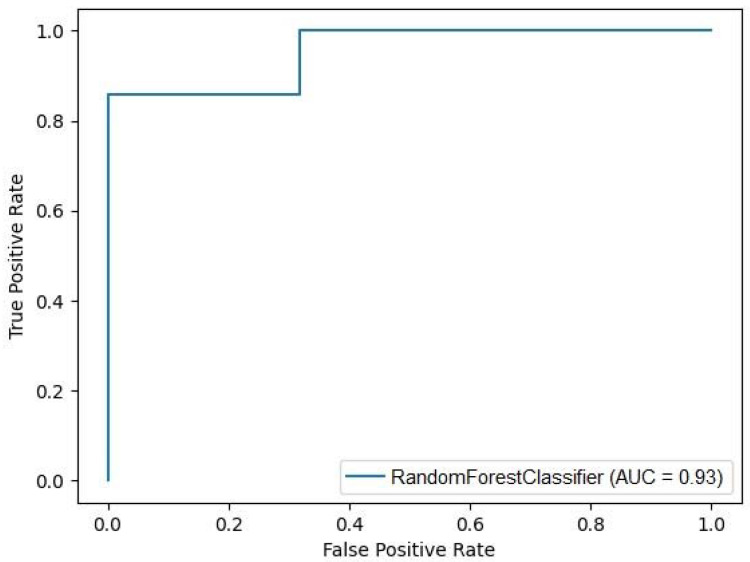
Random Forest Classifier performance illustrated with a receiver–operator curve (ROC) plot. RFC with given input variables (MPV, neutrophil count, and SII) showed a very good ability to discriminate patients with the need to perform dialysis.

**Figure 2:**
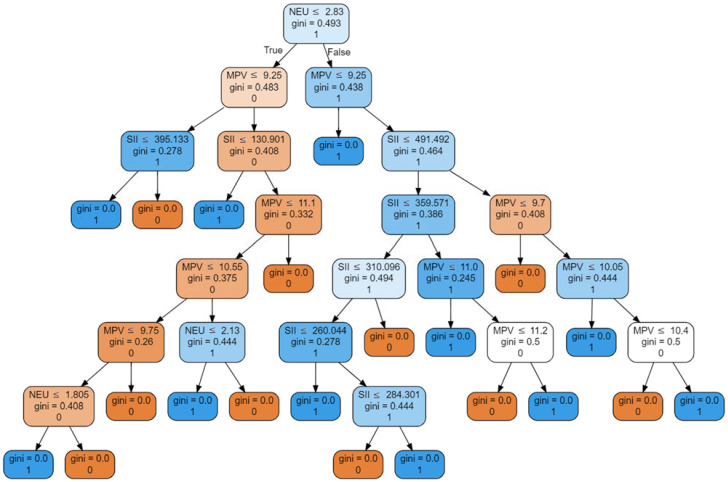

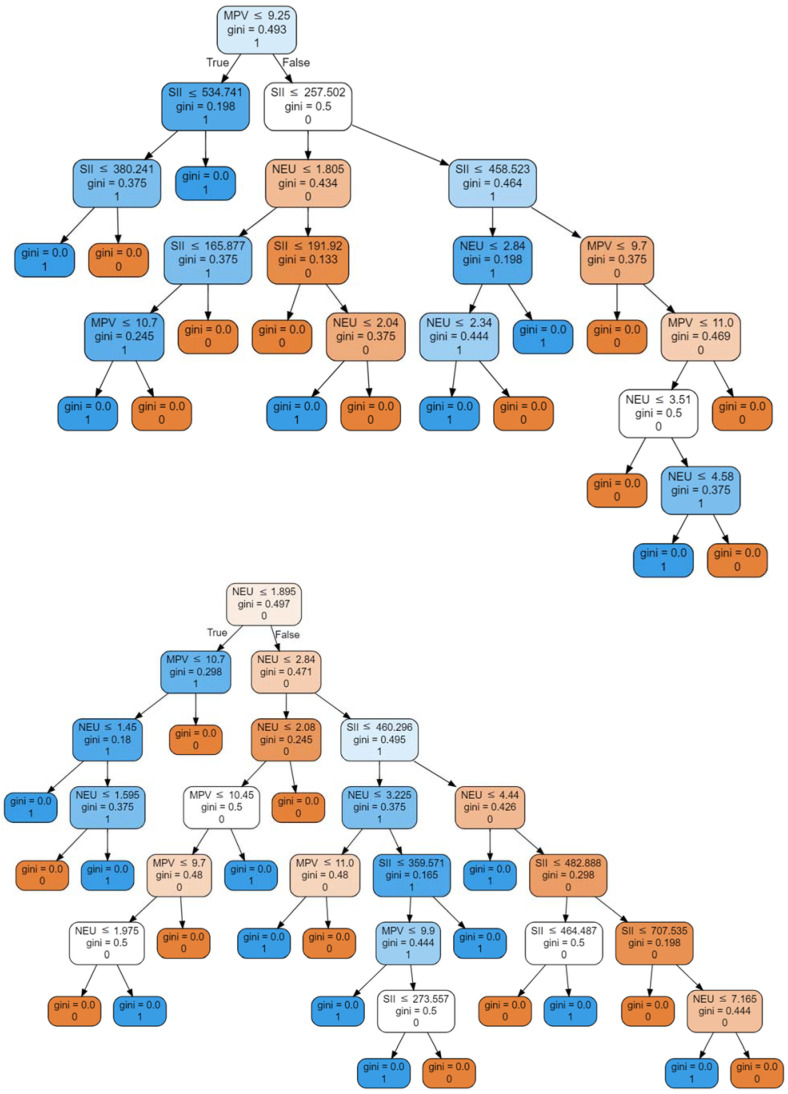
Selected decision trees from the RFC model, with serum neutrophil count, SII, and MPV as input variables, predicting need for hemodialysis. In the illustrated graphs, we move from the top node toward the right or left child of the node. Each node has a condition that, when met, means moving one level lower to the left child node. The value of Gini denotes the impurity index of the node. Each node means dividing a certain set into two, which may contain different numbers of samples marked with specific labels 0 or 1. If, after the division, we obtain a subset containing only samples with one type of label, e.g., 0, this indicator reaches the value 0.0. Each node also contains the dominant label in a given division. The partial results from all trees are collected, and the prevailing result is the final one.

As a result of eliminating the number of input variables and evaluating the resulting models, the best classifying model was obtained with a high discriminating ability of patients requiring dialysis through iteration. The best Random Forest Classifier contained neutrophil count, MPV, and SII as input variables and achieved the following values: AUROC 0.9286, accuracy 93.75%, precision 0.9437, recall 0.9375, and MCC 0.88 (Figure 1). The statistics for each class were as follows: precision 0.90, recall 1.00, and f1-score 0.95 for children with CKD 4–5 on conservative treatment; precision 1.0, recall 0.86, and f1-score 0.92 for patients on chronic dialysis. The values of Gini feature importance measured for MPV, neutrophil count, and SII were 0.295, 0.34, and 0.365, respectively. Exemplary decision trees are shown in Figure 2. 

In the meantime, a similar study conducted for APD and HD patients gave promising results, but due to the small group size, they require further testing on a larger population. The model intended to differentiate patient groups based on the above three parameters (MPV, neutrophil count, and SII) achieved an AUROC of 0.8750, an accuracy of 87.50%, MCC of 0.7745, a precision of 0.9000, and a recall of 0.8750. Therefore, a larger database is required to test its efficiency.

## 5. Discussion

In our study, the RFC model built with the input variables of neutrophil count, MPV, and SII turned out to be the best predictor of the progression of pediatric CKD into end-stage kidney disease requiring dialysis. Out of these variables, SII showed the largest share in the prediction of the need for renal replacement therapy. Therefore, the model constructed with CBC-derived parameters outperformed those based on classical indices of CKD-related complications, like anemia, CKD–mineral-bone disease, or acidosis. These findings underline the importance of chronic inflammation in the deterioration of kidney function, which should not be neglected when compared to the influence of other risk factors. 

The role of persistent low-grade inflammation in adults with CKD has been vastly analyzed and discussed [3]. Chronic inflammation causes dysregulation of intrarenal microcirculation, changes in the distribution of perfusion, and promotion of oxidative stress, resulting in irreversible tubular damage, nephron dropout, fibrosis, and gradual loss of renal function [3,7]. Although research studies in adults reported the elevation of inflammatory biomarkers and linked their increase to CKD progression, inflammation was treated as a bystander rather than the main player [2,3].

Changes in the white blood cell count and proportion observed in adults with CKD included higher granulocyte and monocyte count and lower lymphocyte count [12,13,37]. The latter was concordant with our observation of the decrease in lymphocyte count along with the transition from CKD stage 5 into chronic dialysis. In adult CKD patients, spikes in the percentage of granulocytes and monocytes were identified as independent risk factors for disease progression and death [12]. In addition, higher peripheral neutrophil count was found to be positively and independently associated with the rapid progression of CKD [37]. Despite extensive analysis in the adult CKD population, the number of publications on complete blood count-derived parameters in pediatric CKD patients is limited to one study. Cetın et al. assessed the relationship between the percentage and count of immature granulocytes and inflammation in children with CKD stages 1–4 [38]. They concluded that both parameters had a predictive value for the presence of inflammation in CKD pediatric patients [38].

In our study, no difference was reported in the leukocyte/granulocyte/monocyte count between the pre-dialysis and dialysis groups, although the tendency toward higher monocyte percentage in children on chronic dialysis vs. those with CKD 4–5 was noticeable. Moreover, correlations between all cell counts and indices of toxemia/inflammation were statistically significant.

Consequently, the neutrophil-to-lymphocyte ratio (NLR) was proposed as a supplementary marker of CKD progression and the need for starting hemodialysis [16,39,40]. Even in the patients with normal white blood cell count, higher NLR was associated with higher long-term all-cause mortality in CKD stage 5 [41]. Moreover, the initial white blood cell count seemed to have a long-term impact on the all-cause mortality of adult patients on peritoneal dialysis [1]. Although our patients showed no difference regarding the NLR values in pre-dialysis versus dialysis conditions, NLR correlated with CRP in both subgroups.

Mean platelet volume (MPV), platelet-to-lymphocyte ratio (PLR), and mean platelet volume-to-lymphocyte ratio (MPVLR) are other parameters derived from complete blood count of potential use in the assessment of CKD-related chronic inflammation. The dysfunction of platelets and changes in their functional properties observed in CKD patients indicate the modulating role of platelets in persistent inflammation. Platelets may interfere with the inflammation pathways due to their role in maintaining vascular integrity. Additionally, they are able to influence leukocyte function and monocyte differentiation by cell–platelet adhesion or by the release of soluble mediators, microparticles, and cytokines [17,23]. The increased MPV is linked with CKD progression in adult patients [19]. In our study group, platelet counts were correlated with CRP, whereas MPV showed a connection to albumin and cholecalciferol concentrations. The research by Xu et al. showed higher MPVLR in adults with CKD stages 3–4 compared to those with CKD stages 1–2 and an independent association between higher MPVLR and the presence of inflammation in non-dialysis patients with CKD [20].

In adult CKD patients on maintenance hemodialysis, the combination of NLR, MLR, and PLR was proposed for a more precise assessment of inflammation and all-cause mortality [17,21]. Recently, SII, which combines neutrophil, lymphocyte, and platelet counts, has been suggested as a more comprehensive indicator of systemic inflammation. SII has been studied extensively in different chronic and acute pathologies, including those of the kidney and urinary tract. However, most of the research studies reporting the link between SII and renal diseases were conducted in adults. Qin et al. have shown that higher SII was positively correlated with the risk of increased urinary albumin excretion in adults [25]. SII also seems to have a predictive value in adult patients with uroepithelial cancer. According to the meta-analysis by Jin et al., there is an association between high SII and poor survival outcomes in adult patients with renal cell carcinoma [26]. Similarly, the meta-analysis by Wang et al. has shown that significantly elevated SII was an indicator of poor overall survival in adult patients with renal cell carcinoma, prostate carcinoma, bladder carcinoma, testicular cancer, and urothelial carcinoma [27]. 

The diagnostic value of SII in pediatric nephrology has been investigated in children with urinary tract infections. Karakaya et al. assessed the role of CBC-derived parameters in predicting acute pyelonephritis and showed that SII was among the best predictors of the development of acute pyelonephritis [28]. Likewise, according to Kocaaslan et al., SII might have a predictive value toward renal involvement in newborns with urinary tract infections [29].

The elevated SII has a predictive value for mortality risk among CKD non-dialyzed and hemodialyzed adults [30,31]. Lai et al. reported that the elevated SII value on admission was an independent risk factor for all-cause, cardiovascular, and cancer mortality among non-dialysis CKD patients, followed up for a median of 4.5 years after coronary angiography [31]. The study by Ran et al. not only demonstrated that high SII correlated with shorter long-term survival in hemodialysis patients but also proved that it is an independent risk factor for protein-energy wasting in these patients [30]. SII has also been linked to a higher risk of diabetic kidney disease in adults with type 2 diabetes mellitus [32]. The possible use of SII as a marker of the increased risk of delayed graft function and acute rejection was assessed by Halpern et al. in the population of adult kidney transplant recipients [33]. Their findings showed the limited utility of SII as an independent predictor of outcome after kidney transplantation. However, the authors stated that SII might be useful if combined with other known risk factors for poor prognosis in post-transplant patients [33]. 

Contrary to the adult population, pediatric patients with CKD were not tested for the SII values and their prognostic abilities. Thus, to the best of our knowledge, this study is the first to assess the link between SII and the progression of CKD in pediatric patients. SII in both subgroups correlated with CRP, alkaline phosphatase, serum albumin, and pH of blood gases. 

The computer program has been equipped with a simple heuristic algorithm that allows for optimally finding the best predictive model and flexibly scaling solutions to larger databases in other centers. The brute-force solution requires enormous computational complexity, although once the process of parallelization is introduced, the following stages do not cause particular problems. However, in other, broader applications, it is possible to speed up the evaluation of models and secure more effective adaptation of classifiers to growing data sets. We have empirically demonstrated the effectiveness of optimization techniques based on heuristics as opposed to previous brute-force applications [42]. 

Moreover, it is possible to scale the developed models to new data, and this perspective seems essential in light of our preliminary results on the prediction of the type of dialysis (APD vs. HD) that requires testing on a larger dataset. 

Another step is the analysis of risk factors, with an emphasis on the use of Gini feature importance as a parameter indicating critical factors for classifying patients and discrimination in risk models. Other authors mention its use in the assessment of unrelated and COVID-19-related mortality in dialysis patients [43,44]. It was also used in predicting the outcome in patients with lupus nephritis [42] and in estimating graft function in patients after kidney transplantation [45]. 

We also have to acknowledge the limitations of our study. First, the size of the subject is limited by the small number of data and by the fact that it was conducted in a single center. Second, we evaluated only selected markers of inflammation and a limited number of parameters reflecting other CKD-related complications. Thus, this analysis did not cover all aspects of multilayer connections between inflammatory indices, markers of CKD, and blood cell count-derived ratios. 

## 6. Conclusions

Complete blood cell count-driven parameters provide simple and cost-effective insight into the inflammatory status of children with chronic kidney disease. Our study showed that the CBC-driven parameters, particularly neutrophil count, mean platelet volume, and systemic immune inflammation index, might be of added value while predicting the need for chronic dialysis in pediatric CKD patients. However, further studies in that field are required to assess their potential role in clinical practice. 

## Figures and Tables

**Table 1:** Main correlations between CBC-derived ratios and examined parameters in the whole study group.

CBC-Derived Ratios	Body Weight	CRP	Alkaline Phosphatase	Serum Albumin	Serum Creatinine	eGFR
SII	R = 0.19	R = 0.31	R = −0.38	R = −0.27	R = −0.02	R = −0.012
	*p* = 0.11	*p* = **0.014**	*p* = **0.004**	*p* = **0.039**	*p* = 0.84	*p* = 0.92
NLR	R = 0.35	R = 0.31	R = −0.44	R = −0.15	R = −0.003	R = 0.06
	*p* = **0.003**	*p* = **0.013**	*p* = **0.0007**	*p* = 0.23	*p* = 0.98	*p* = 0.63
LMR	R = −0.3	R = −0.06	R = 0.16	R = 0.12	R = −0.14	R = 0.013
	*p* = **0.015**	*p* = 0.64	*p* = 0.22	*p* = 0.36	*p* = 0.24	*p* = 0.91
PLR	R = 0.26	R = 0.14	R = −0.41	R = −0.32	R = 0.15	R = −0.12
	*p* = **0.035**	*p* = 0.29	*p* = **0.002**	*p* = **0.012**	*p* = 0.22	*p* = 0.32

CBC—complete blood cell count; SII—systemic immune inflammation index; NLR—neutrophil-to-lymphocyte ratio; LMR—lymphocyte-to-monocyte ratio; PLR—platelet-to-lymphocyte ratio; CRP—C-reactive protein; R—Spearman’s rank correlation coefficient; eGFR—estimated glomerular filtration rate; *p* < 0.05 assumed statistically significant and put in bold.

**Table 2:** Basic characteristics of the training and testing groups.

Feature	Training Set Mean ± SD (Min–Max) *n* = 52	Testing Set Mean ± SD (Min–Max) *n* = 14
Gender—male	30 (57.7%)	6 (42.9%)
Gender—female	22 (42.3%)	8 (57.1%)
sCr (mg/dL) *	5.94 ± 2.8 (1.38–15.05)	7.75 ± 3.74 (2.76–16.24)
WBC (×10^3^/uL)	6.9 ± 2.31 (2.6–14.05)	6.32 ± 1.7 (4.1–11.16)
PLT (×10^3^/uL)	236.29 ± 81.64 (57–513)	221.86 ± 48.10 (163–334)
MPV (fL)	10.03 ± 0.85 (8.2–12.4)	10.16 ± 1.12 (8.8–11.8)
NEU (×10^3^/uL)	3.62 ± 1.61 (1.09–8.8)	3.16 ± 1.15 (1.17–4.97)
LYM (×10^3^/uL)	2.33 ± 0.96 (0.72–6.6)	2.21 ± 1.01 (1–4.75)
MON (×10^3^/uL)	0.53 ± 0.18 (0.27–1.07)	0.85 ± 1.19 (0.35–4.9)
CRP (mg/L)	2.65 ± 6.77 (0.1–35.75)	1.67 ± 2.98 (0.09–10.95)
SII	389.21 ± 252.44 (77.34–1582.13)	361.47 ± 202.14 (136.99–891.7)
PLR	109.80 ± 40.51 (46.34–276.79)	113.46 ± 38.73 (54.53–185)
NLR	1.67 ± 0.9 (0.4–4.68)	1.7 ± 1.11 (0.62–4.82)
LMR	4.59 ± 1.66 (1–8.05)	3.96 ± 1.74 (0.42–7.68)
CKD 4 *	9 (17.3%)	2 (14.3%)
CKD 5 *	11 (21.2%)	5 (35.7%)
Dialysis–target	32 (61.5%)	7 (50.0%)
HD *	18 (34.6%)	2 (14.3%)
APD *	14 (26.9%)	5 (35.7%)

SD—standard deviation, min—minimal value, max—maximal value, sCr—serum creatinine, WBC—white blood cell count, PLT—platelet count, MPV—mean platelet volume, NEU—neutrophil count, LYM—lymphocyte count, MON—monocyte count, CRP—C-reactive protein, SII—systemic immune-inflammation index, PLR—platelet-to-lymphocyte ratio, NLR—neutrophil-to-lymphocyte ratio, LMR—lymphocyte-to-monocyte ratio, CKD 4—chronic kidney disease stage 4, CKD 5—chronic kidney disease stage 5, HD—hemodialysis, APD—automated peritoneal dialysis, *—not included in developing classifier due to the oversimplification and direct implications to predicting value.

## Data Availability

The datasets generated and analyzed during the current study are available from the corresponding author upon reasonable request.
